# The short-term effect of particulate matter on tuberculosis: a time-series study in southern China

**DOI:** 10.1186/s12879-026-13369-5

**Published:** 2026-05-08

**Authors:** Yuhui Chen, Shanshan Huang, Lilian Zeng, Guanhai Li, Keer Ou, Huiying Feng, Xing Li, Anqi Liang, Jianpeng Xiao, Liang Chen, Jiawen Wang

**Affiliations:** 1 Public Health Medical Center of Guangdong Province, Guangzhou, 510630 China; 2 Guangdong Provincial Institute of Public Health, Guangdong Provincial Center for Disease Control and Prevention, Guangzhou, 511430 China; 3 Guangzhou Chest Hospital, Guangzhou, 510095 China

**Keywords:** Particulate matter, Tuberculosis, Risk, Time-series analysis

## Abstract

**Background:**

Although increasing evidence suggests an association between particulate matter (PM) and tuberculosis (TB), epidemiological studies in southern China remain limited.

**Methods:**

Daily tuberculosis (TB) case numbers, air pollution, and meteorological data for four cities (Guangzhou, Shenzhen, Foshan and Jiangmen) in southern China during 2015–2019 were collected. A two-stage analytical approach was used to evaluate the association between PM and TB incidence, with stratification by sex, age, disease severity, and city.

**Results:**

A total of 101,567 TB cases were reported in four cities. The relationships between PM_2.5_ and PM_10_ and TB incidence were approximately linear, with the highest risk observed at a cumulative lag of 0–2 days (lag02). Each 10ug/m^3^ increase in PM_2.5_ was associated with a 0.84% (95%CI: 0.28%,1.41%) increase in TB incidence, which exceeded the corresponding risk for PM_10_ (0.71%, 95%CI: 0.26%, 1.15%). The effect of PM_10_ and PM_2.5_ on TB incidence appeared to be greater among females, younger individuals, patients with non-severe TB, and residents of Guangzhou and Shenzhen.

**Conclusions:**

Short-term exposure to ambient PM may significantly increase TB incidence risk in southern China. These findings provide scientific evidence to support the development of integrated TB prevention and air quality management strategies.

**Clinical trial number:**

Not applicable.

**Supplementary Information:**

The online version contains supplementary material available at 10.1186/s12879-026-13369-5.

## Background

Tuberculosis (TB) is an infectious disease caused by *Mycobacterium tuberculosis* that primarily affects the lungs and lymphatic system. According to the World Health Organization (WHO), approximately 10.6 million TB cases occurred worldwide in 2021 [1]. Despite declining trend in TB incidence and mortality over the past 30 years, China remains among the countries with the highest TB burden [2]. In 2021, China reported nearly 640,000 cases of tuberculosis, ranking third among 40 notifiable infectious diseases [3].

Previous studies indicated that TB results from a combination of multiple etiological factors, including nutritional status, HIV infection, population mobility, diabetes and lifestyle [4–7]. In recent years, a number of epidemiological studies have found that short-term exposure to air pollutants may also be strongly associated with the development of TB [8, 9], particularly ambient particulate matter (PM) [10–12]. For example, a study in Wuhan reported a 17.03% increase in TB incidence per 10 µg/m³ increase in PM_2.5_ at a 7-day lag [13]. However, the existing evidence remains inconsistent. A systematic review and meta-analysis of 17 studies found no significant association between ambient PM and TB risk [14]. This inconsistency may stem from methodological heterogeneity across studies, most of which employed single-city designs that limit the comparability and generalizability of findings. To address this limitation, multi-city studies utilizing standardized analytical frameworks are needed to generate more robust and consistent epidemiological evidence.

Southern China, characterized by high population mobility and density, bears a substantial TB burden that ranks among the highest in the country, representing an urgent public health challenge. For example, Guangdong Province reported 66,261 TB cases in 2019, accounting for 8.5% of the cases in mainland China [15]. Additionally, southern China possesses unique environmental and climatic characteristics, including a subtropical monsoon climate with year-round warm and humid conditions, which may influence both PM composition and TB transmission dynamics. However, epidemiological evidence on the PM-TB relationship in this region remains limited, with most previous studies focusing on central, northern, or western China [12, 16, 17]. Therefore, investigating the impact of ambient PM on TB in South China is of considerable importance for developing region-specific public health strategies.

In this study, we employed a multi-city time-series design to examine the association between short-term exposure to ambient PM and TB incidence in four cities in Guangdong Province, South China. We further explored the effects of ambient PM across different subgroups defined by sex, age, and disease severity. The findings from this study may provide scientific evidence for the formulation of targeted TB prevention and control strategies, and support environmental health policies aimed at reducing the health burden attributable to air pollution.

## Methods

### Study area

This study was conducted in four cities in Guangdong Province, including Guangzhou, Shenzhen, Foshan and Jiangmen (Figure S1). Guangdong Province is located on the southern coast of China, adjacent to Hong Kong and Macau.

The selection of these four cities was based on the following considerations: (1) all four cities maintain complete, high-quality TB surveillance and air quality monitoring data throughout the study period, with sufficient daily TB cases for city-specific time-series analyses; (2) the four cities represent diverse urban types—Guangzhou (provincial capital, population 15.31 million), Shenzhen (special economic zone, 13.44 million), Foshan (medium-sized city, 8.16 million), and Jiangmen (smaller city, 4.75 million)—together accounting for 36% of Guangdong’s population; and (3) all four cities share a subtropical monsoon climate with warm temperatures and high humidity year-round, which reduces meteorological confounding and enhances the internal consistency of pooled estimates.

### Data collection

Tuberculosis is classified as a notifiable Class B infectious disease in China, requiring cases to be reported to health authorities within 24 h of detection through the Chinese Tuberculosis Information Management System. Reported information includes age, sex, address, date of symptom onset, date of treatment initiation, etiological examination results, and treatment plan. For this study, TB data from 2015 to 2019 were collected from the Center for Tuberculosis Control of Guangdong Province, and aggregated into daily counts based on the date of symptom onset. The tuberculosis cases were also grouped by sex (male and female), age (0–64 years, 65 years and over) and severity (general and serious).

Daily 24-hour mean concentrations of ambient air pollutants in the four cities during the study period were obtained from the National Urban Air Quality Real-time Publishing Platform(https://air.cnemc.cn:18007/), including particulate matter with an aerodynamic diameter of 10 μm or less (PM_10_), particulate matter with an aerodynamic diameter of 2.5 μm or less (PM_2.5_), Nitrogen dioxide (NO_2_), Sulfur dioxide (SO_2_), carbon monoxide (CO) and Ozone (O_3_).

Daily 24-hour meteorological monitoring data for the same period were obtained from the China Meteorological Data Sharing Service (http://data.cma.cn/), including mean temperature(°C) and relative humidity (%) for each city.

Data on the registered population, permanent resident population, and population density for the four cities in 2019 were obtained from the *Guangdong Statistical Yearbook 2020*. The floating population was calculated as the difference between the permanent resident population and the registered population.

### Statistical analysis

A two-stage analytical approach was employed to evaluate the association of short-term exposure to PM and TB risk in this study. In the first stage, we fitted an over-dispersed generalized additive Poisson model (GAM) for each city, given that TB case counts followed an approximate quasi-Poisson distribution. We initially applied a smoothing spline function to the PM variable to estimate the exposure-response relationship curves. The linearity of the association was evaluated by both visual inspection of the spline curves and the likelihood ratio test comparing models with and without non-linear spline terms. A *P*-value for non-linear > 0.05 was considered to support a linear relationship. To ensure methodological consistency and facilitate direct comparison of effect estimates across cities, when at least half of the cities (≥ 50%) exhibited linear relationships, linear terms were uniformly applied to all cities for subsequent analysis. For covariates, we included days of the week (DOW) and public holidays (PH) variables to control for weekly exposure patterns and holiday effects. We also used smoothing spline functions incorporating calendar time, daily temperature and relative humidity to adjust for seasonal patterns, long-term trends and meteorological factors while accounting for potential non-linear confounding. Briefly, the model formula can be expressed as:

$$\eqalign{ {\rm{Log}}\left({E({Y_t}} \right) & = s\left({P{M_t}} \right) + DOW + PH + s\left({day} \right) \cr & + s\left({T{m_t}} \right) + s\left({R{H_t}} \right) + \alpha \cr}$$

where *E(Y)* denotes the expected number of daily TB count on day t; *s( )* represents smoothing spline function for nonlinear variables; *PM* denotes the daily PM concentration (PM_10_ or PM_2.5_); *day* indicates the calendar time variable; and *α* is the intercept for the model. Based on previous studies [12, 16] and minimization of the Akaike information criterion (AIC), we applied 7 degrees of freedom(*df*) per year for temporal trends and 3 *df* for particulate matter, temperature, and relative humidity.

In the second stage, a meta-analysis with random effect was used to pool city-specific estimates and obtain overall effect estimates for southern China. If the PM-TB incidence association was considered linear or approximately linear, results were presented by the indicator of excess risk (ER) with 95% confidence interval (CI), representing the percentage change in TB incidence risk for per 10 ug/m^3^ increase in daily PM concentration. We further performed stratified analyses by sex, age and severity. Additionally, we constructed linear regression models to examine whether city-level population mobility and population density influenced the PM–TB association, using ER as the dependent variable.

Given the potential delayed health effects of air pollutants, we examined the lag effects from the current day(lag0) through 14 lagged days (lag1, lag2, …, lag14). We also assessed cumulative effects using moving averages from the current day through the previous 1 to 14 days (lag01, lag02, …, lag014). To account for potential confounding by co-pollutants, we further conducted a series of dual pollutant models for PM_2.5_ and PM_10_. Prior to conducting dual pollutant models, we calculated variance inflation factors (VIF) to assess multicollinearity among pollutants. All VIF values were below 5 (Table S1), indicating acceptable collinearity levels. Therefore, all co-pollutants (SO_2_, NO_2_, CO, and O_3_) were included in the dual pollutant models.

To test the robustness of our findings, we performed a series of sensitivity analysis. First, we changed the *df* of the smoothing spline function from 6 to 9 per year for calendar time. Then, we adjusted the *df* of temperature and relative humidity variables from 3 to 6.

All data were conducted using R project software (version 4.0.2). The “mgcv” and “metafor” package were used for model fitting and meta-analysis, respectively. All figures were made using “ggplot2” package. The results of the statistical tests were two-sided with values of *P* < 0.05 as statistical significance.

## Results

### Basic characteristics of the study sample

Table 1 presents the average daily number of TB cases, air pollutant concentrations and meteorological factors during the study period. From 2015 to 2019, a total of 101,567 TB cases were reported across the four cities, with 47,319 in Guangzhou, 23,245 in Shenzhen, 16,725 in Foshan and 14,278 in Jiangmen. The daily time series of TB cases is depicted in Figure S2. The number of TB cases was greater in males (70255, 69.17%) than in females (31312, 30.83%), cases aged 0–64 years accounting for 86.85% (88212) of all cases, and 91.86% (93295) were classified as general (non-severe) cases. During the study period, daily PM_10_ concentration ranged from 8.43 ug/m^3^ to 268.54 ug/m^3^ with mean of 55.59 ug/m^3^, and PM_2.5_ ranged from 4.02 ug/m^3^ to 227.06 ug/m^3^ with mean of 34.39 ug/m^3^. The four cities have a combined floating population of 18.088 million, with an average population density of 1829.84 persons/km^2^.

**Table 1:** Characteristics of the TB cases and environmental variables in four cities, 2015–2019

Variable	Guangzhou	Shenzhen	Foshan	Jiangmen	Total
**Tuberculosis** [Median (IQR)]					
Total	21 (13)	12 (8)	8 (5)	7 (6)	11 (10)
Sex					
Male	15 (10)	8 (6)	6 (4)	5 (5)	7 (8)
Female	7 (6)	4 (4)	2 (3)	1 (2)	3 (4)
Age(years)					
0–64	19 (12)	11 (7)	7 (5)	5 (5)	9 (10)
≥ 65	3 (4)	0 (1)	1 (2)	1 (2)	1 (2)
Severity					
General	20 (12)	10 (7)	7 (5)	6 (5)	10 (10)
Serious	1 (2)	0 (2)	1 (2)	0 (1)	1 (2)
**Environmental Factor** (Mean ± SD)					
PM_2.5_	34.39 ± 18.97	26.81 ± 14.38	36.22 ± 21.19	32.46 ± 20.03	32.47 ± 19.14
PM_10_	55.59 ± 26.73	44.53 ± 21.65	59.31 ± 30.66	56.21 ± 29.08	53.91 ± 27.80
SO_2_	10.52 ± 4.38	7.38 ± 2.29	12.76 ± 6.06	11.13 ± 6.09	10.45 ± 5.32
NO_2_	45.5 ± 18.34	29.81 ± 10.75	41.32 ± 18.68	33.7 ± 19.33	37.58 ± 18.20
CO	0.87 ± 0.21	0.74 ± 0.19	0.84 ± 0.22	0.84 ± 0.25	0.82 ± 0.23
O_3_	48.98 ± 26.78	60.72 ± 25.99	50.22 ± 28.71	52.7 ± 32.44	53.16 ± 28.93
Temperature	22.12 ± 6.00	23.12 ± 5.52	22.74 ± 5.96	23.4 ± 5.54	22.85 ± 5.78
Relative humidity	79.63 ± 10.75	78.62 ± 11.18	80.2 ± 10.47	80.11 ± 11.49	79.64 ± 10.99
**Demographic variables**					
Floating population (×100000)	57.69	80.21	35.46	7.52	180.88
Population density (/km^2^)	6141	6484	2145	487	1829.84

*IQR: Interquartile Range; SD: Standard Deviation

### Correlation analysis of environmental factors

**Fig. 1:**
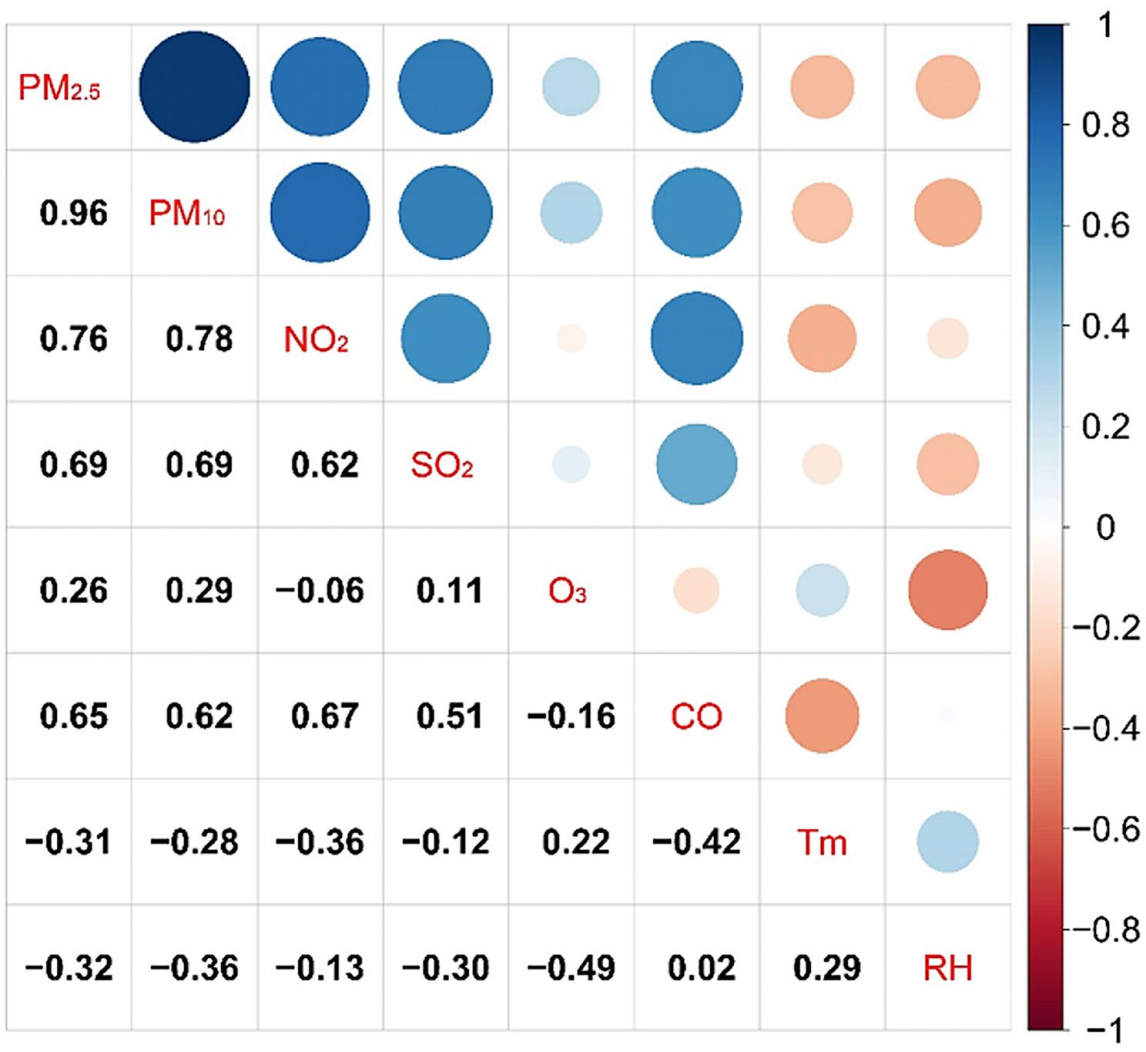
Spearman’s correlation coefficient for air pollutants and meteorological factors in four cities

Figure 1 displays the Spearman correlation coefficients among air pollutants and meteorological factors. Daily PM_2.5_ and PM_10_ concentrations were both moderately and positively correlated with other pollutants, except with O_3_ which was weak and positive correlation. In addition, ambient PM concentration was weakly and negatively correlated with meteorological factors.

### Association between ambient particulate matter and tuberculosis risk

**Fig. 2:**
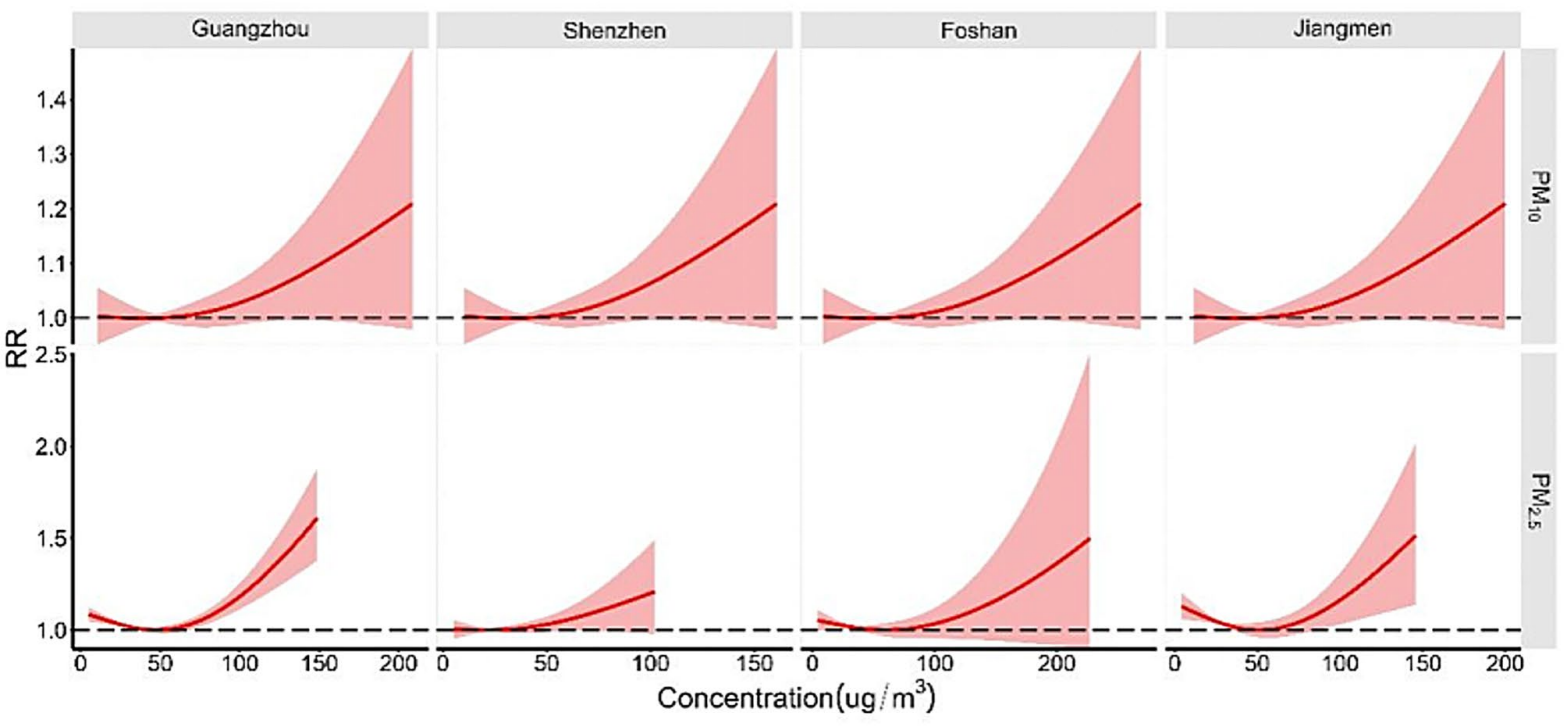
The exposure-response curves of ambient PM_10_ and PM_2.5_ concentrations(ug/m^3^) with risk of tuberculosis

**Fig. 3:**
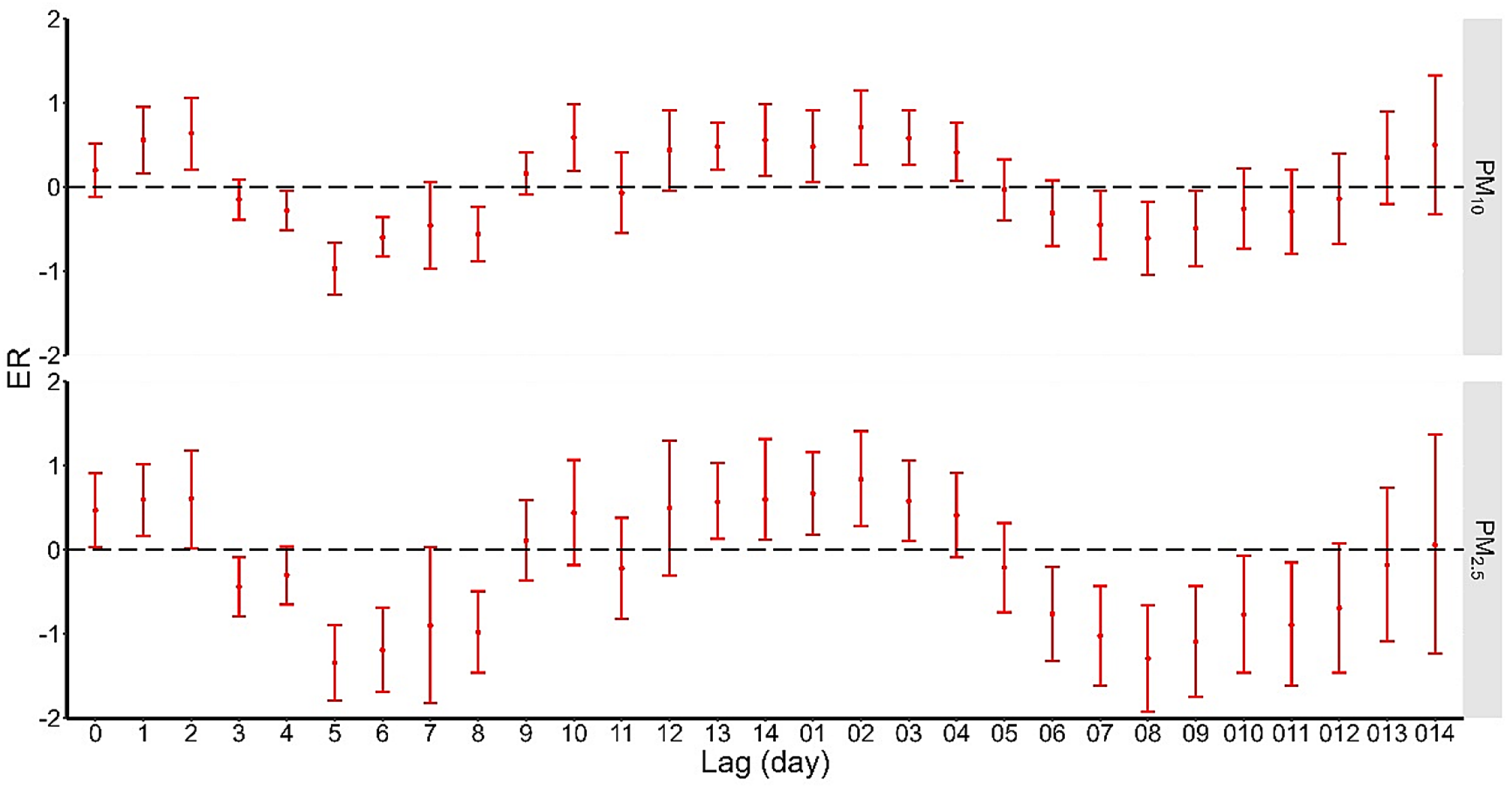
Excess risk(%,95%CI) of TB incidence associated with per 10ug/m^3^ increase in PM concentrations

We examined the exposure-response relationships between current-day (lag0) PM_10_ and PM_2.5_ concentrations and tuberculosis incidence across the four cities (Fig. 2). Although statistically significant deviations from non-linearity were detected in Guangzhou and Foshan (Table S2), the overall patterns consistently showed monotonically increasing trends. Given this approximately linear relationship, we adopted linear models to compare the effects across different single-day lags and cumulative lag periods up to 14 days (Fig. 3). Among the single-day lags, TB incidence risk for both PM_2.5_ or PM_10_ were highest for a 2-day lag(lag2), with significant heterogeneity across cities (PM_2.5_: I^2^ = 55.88%, *P* < 0.01; PM_10_: I^2^ = 61.86%, *P* < 0.01). Among cumulative lags, the highest risks were observed at 0–2 days (lag02), with significant location heterogeneity (PM_2.5_: I^2^ = 30.24%, *P* < 0.01; PM_10_: I^2^ = 45.58%, *P* < 0.01).

**Fig. 4:**
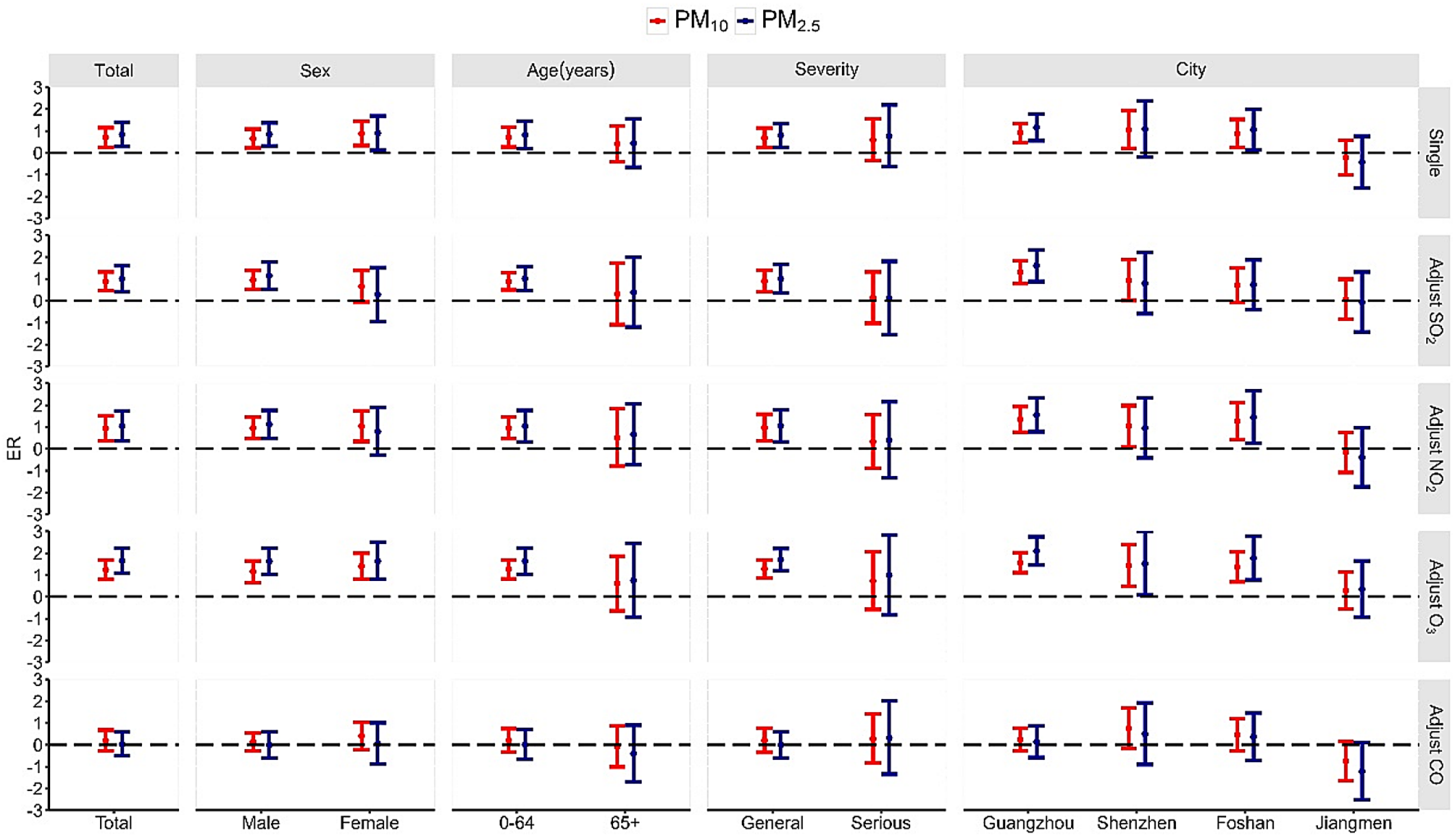
Excess risk(%,95%CI) of cumulative 2-day PM concentrations for the single- and two-pollutant models

Overall, we observed a 0.84% (95% CI: 0.28%, 1.41%) increase for TB incidence for per 10ug/m^3^ increase in PM_2.5_ concentration, which was higher than the risk for PM_10_(0.71%, 95% CI: 0.26%, 1.15%). In the two-pollutant model including SO_2_, NO_2_, O_3_, or CO, neither PM_10_ nor PM_2.5_ showed statistically significant associations with TB after adjustment for CO. In models adjusted for SO_2_, NO_2_ and O_3_, the estimated risks for both PM_10_ and PM_2.5_ were enhanced and remained significant. Notably, after adjusting O_3_, the excess risk of TB incidence per 10ug/m^3^ increase in PM_2.5_ and PM_10_ increased to 1.65% (95% CI: 1.07%, 2.23%) and 1.24% (95% CI: 0.79%, 1.70%), respectively (Fig. 4).

In the subgroup analyses, females exhibited slightly higher PM-associated TB risk than males; younger individuals (0–64 years) had higher risk than older individuals (≥ 65 years); and patients with non-severe TB had higher risk than those with severe disease. The associations were not statistically significant among older individuals or patients with severe disease. Geographically, the effects of PM on TB incidence were strongest in Guangzhou and Shenzhen, whereas the association in Jiangmen was negative but not statistically significant. Furthermore, PM_2.5_ showed higher risk estimates than PM_10_ across all subgroups.

We further examined whether demographic variables influenced PM-associated TB risk (Table 2). Both population mobility and population density showed positive associations with excess risk; however, these associations did not reach statistical significance (*P* > 0.05).

**Table 2:** The impact of demographic variables on excess risk of cumulative 2-day PM concentrations

Variable	PM_10_		PM_2.5_	
	β	*P*	β	*P*
Floating population (×100000)	0.016	0.128	0.020	0.183
Population density (/km2)	0.16 × 10^− 3^	0.203	0.20 × 10^− 3^	0.229

### Sensitivity analysis

Sensitivity analyses demonstrated that varying the *df* for temporal trends from 6 to 9 per year did not substantially alter the estimated risks for PM_10_ or PM_2.5_ at the 2-day cumulative lag (Table 3). Similarly, varying the *df* for meteorological factors from 3 to 6 yielded consistent results.

**Table 3:** Excess risk(%,95% CI) of TB incidence by sensitivity analysis

Item	PM_10_	PM_2.5_
*df* value for temporal trends		
6	0.72 (0.32, 1.12)	0.83 (0.33, 1.34)
7	0.71 (0.26, 1.15)	0.84 (0.28, 1.41)
8	0.76 (0.31, 1.21)	0.86 (0.37, 1.46)
9	0.75 (0.30, 1.20)	0.86 (0.35, 1.49)
*df* of meteorological conditions		
3	0.71 (0.26, 1.15)	0.84 (0.28, 1.41)
4	0.71 (0.27, 1.16)	0.85 (0.29, 1.41)
5	0.71 (0.27, 1.15)	0.85 (0.28, 1.41)
6	0.73 (0.30, 1.16)	0.86 (0.39, 1.42)

## Discussion

This study contributes to the epidemiological evidence on the relationship between ambient PM and TB incidence in South China. Our findings indicate that short-term exposure to ambient PM is associated with an increased TB incidence risk, with stronger effects observed for PM_2.5_ compared to PM_10_. Subgroup analyses identified females, younger individuals, and patients with non-severe TB as particularly susceptible populations.

Several previous studies have also reported positive associations between PM and TB risk [12, 16–18]. A study in Chengdu, China, used a distributed lag nonlinear model, found a cumulative relative risk of 1.06 (95% CI: 1.03, 1.09) for every 10ug/m^3^ in PM_10_ above the GB3095-2012 annual standard (70 µg/m³) over a 0–21 day lag [18]. Another study in Wulumuqi found that each 1 mg/m^3^ increase in the monthly average PM_10_ and PM_2.5_ concentrations was associated with 0.08% and 0.09% increases in pulmonary TB risk, respectively [17]. A multi-city study in Anhui Province also observed positive associations between ambient PM and TB hospitalization [12]. However, the magnitude and significance of associations reported vary considerably across studies, and some studies found no significant relationship [13]. These inconsistencies may stem from several factors, including differences in climatic conditions, PM sources and composition, population susceptibility, TB case definitions (incidence, hospitalization or mortality), and methodological choices regarding temporal resolution, lag structures, and confounding adjustment.

Previous studies have reported inconsistent findings regarding the PM–TB relationship after adjustment for co-pollutants [14, 19, 20]. In present study, we adjusted for non-highly correlated air pollutants of PM_10_ and PM_2.5_ by the two-pollutant model, and found that adjusting for SO_2_ and O_3_ enhanced the risk of PM on TB incidence. Although quantifying the independent effect of a single pollutant is challenging, the increased PM risk estimates observed after adjusted for other pollutants suggest that the PM–TB association warrants attention.

The biological plausibility of our findings is supported by several mechanisms. First, PM inhalation can damage lungs and compromise respiratory immunity through oxidative stress and nitrosative stress pathways [21]. Concurrently, metal ions and other components adsorbed onto PM can enter the body, induce inflammation, and impair lung immune function [8, 21]. Second, PM, especially fine PM, may deposit in the airways and bronchial, triggering cellular defense responses and oxidative stress [22, 23]. Finally, PM-induced physiological responses may interfere with inflammatory signaling pathways, thereby impairing anti-*Mycobacterium tuberculosis* T-cell function [24]. However, the biological mechanisms underlying PM effects on TB remain incompletely characterized, and in-depth research should be carried out in the future to fully characterize the role of PM on tuberculosis.

We observed spatial heterogeneity in PM-induced TB risk, with higher risks identified in Guangzhou and Shenzhen. This discrepancy may be driven by the cities’ status as the most populous centers in Guangdong Province [25], where high-volume population migration facilitates TB spread. The effect is pronounced in Shenzhen, an economic hub with a massive migrant population. Many of these individuals migrate from high-burden TB areas and live in overcrowded settings, creating conditions conducive to higher infection rates despite lower ambient pollution. We therefore examined associations between PM–TB risk and city-level population mobility and density. Although these associations did not reach statistical significance, they exhibited a positive trend (Table 2). Additionally, the chemical composition of PM may vary across cities; specific emission sources in Shenzhen (including vehicle emissions, industrial discharge, and ship exhaust) may generate PM with higher toxicity per unit mass [26]. Finally, the relatively high ozone concentration in Shenzhen may exert a synergistic effect with PM, thereby exacerbating respiratory health impacts.

The sex differences in the effects of PM on TB incidence remain controversial. Although several studies indicate higher risk among males [12, 16, 27], some studies have found that females to be more susceptible [18], consistent with our findings. Several mechanisms may explain this female vulnerability. First, women in China often spend more time cooking indoors and may be exposed to higher concentrations of indoor PM, which synergistically interacts with ambient PM exposure [28]. Second, females typically exhibit greater airway responsiveness to air pollutants and have smaller airway caliber relative to lung size, leading to higher particle deposition [29]. Third, TB symptoms in females may initially be attributed to other conditions, resulting in diagnostic delays during which continued PM exposure may exacerbate disease progression [30]. Notably, while females showed higher PM-associated risk, males have higher absolute TB incidence rates, suggesting that different mechanisms may underlie overall TB risk versus PM-specific effects. Future studies with detailed individual-level exposure data are needed to further elucidate these sex-specific mechanisms. Regarding age distribution, risk was higher among younger individuals (0–64 years). This may reflect greater occupational and environmental PM exposures among working-age adults compared to retirees who spend more time indoors. Additionally, younger adults may mount more pronounced inflammatory responses to PM [31], and higher TB transmission rates in congregate settings (e.g., workplaces, schools) may interact with PM effects to amplify risk. However, our study lacked individual-level exposure and activity data, and future research with personal exposure monitoring is needed to clarify these age-specific mechanisms.

Our findings indicated that the association between PM exposure and TB incidence was more pronounced among non-severe patients compared to severe cases. While the underlying mechanisms remain unclear, several speculative explanations may be considered. One possibility is that PM may primarily affect early-stage TB development rather than severity determinants. Alternatively, survival bias may contribute to this pattern, as severe TB patients often have dominant comorbidities or compromised immune status that could mask the relatively modest effects of PM exposure. It is also possible that severe patients have reduced outdoor activity due to debilitating symptoms, resulting in lower ambient PM exposure prior to diagnosis. However, these explanations are hypothetical and lack direct supporting evidence. Further research with prospective designs, individual-level exposure assessment, and detailed clinical phenotyping is warranted.

This study has several strengths. First, we focused on southern China, an understudied region with high TB burden and unique climatic characteristics, thereby filling an important gap in the literature. Second, the multi-city design with random-effects meta-analysis provides more generalizable estimates than single-city studies. Third, the parallel comparison of PM_2.5_ and PM_10_ effects, systematic lag analysis, dual-pollutant modeling, and comprehensive subgroup analyses collectively provide a thorough characterization of the PM-TB relationship. These methodological features distinguish our study from previous research and strengthen the evidence base for public health decision-making. However, several limitations should be acknowledged. First, the ecological design is useful for generating etiological hypotheses but requires caution in causal inference. Second, we used city-level average pollutant concentrations as proxies for individual exposure, which may introduce exposure misclassification. Third, due to data unavailability and to avoid the confounding effects of the COVID-19 pandemic, TB cases were collected during 2015–2019, and dates of symptom onset were largely self-reported, potentially introducing recall bias. Fourth, the interaction of meteorological factors and other air pollutants with ambient PM should be further investigated in future studies. Finally, data on influenza and other acute respiratory infections (ARIs) were unavailable for inclusion as covariates, potentially introducing residual confounding. Influenza and ARIs could confound the PM-TB association because they share seasonal patterns with PM concentrations (both peaking during cold seasons) and may independently affect TB risk by compromising pulmonary immune defenses or triggering reactivation of latent TB infection. This could potentially lead to overestimation of the observed PM-TB association.

## Conclusions

In summary, this study demonstrates that short-term exposure to ambient PM may significantly elevate TB incidence risk in South China, with PM_2.5_ showing a stronger effect than PM_10_. Females, younger individuals, and patients with non-severe TB appear particularly susceptible. These findings fill an important gap in the epidemiological evidence from southern China and provide scientific support for integrating air quality management into TB prevention and control strategies. Public health interventions targeting susceptible populations during high pollution episodes may help reduce the TB burden attributable to ambient PM exposure.

## Electronic Supplementary Material

Below is the link to the electronic supplementary material.

Supplementary Material 1

## Data Availability

The datasets used and analyzed during the current study are available from the corresponding author on reasonable request.

## Abbreviations

TB: Tuberculosis
PM: Particulate matter
PM_10_: Particulate matter with an aerodynamic diameter of 10 μm or less
PM_2.5_: Particulate matter with an aerodynamic diameter of 2.5 μm or less
NO_2_: Nitrogen dioxide
SO_2_: Sulfur dioxide
CO: Carbon monoxide
O_3_: Ozone
AIC: Akaike information criterion
ER: Excess risk
95%CI: 95% confidence interval
