# Supplementary material for: The short-term effect of particulate matter on tuberculosis: a time-series study in southern China
Source: BMC Infect Dis. 2026 May 8;26:1216. doi: 10.1186/s12879-026-13369-5 (PMC13326357; doi:10.1186/s12879-026-13369-5)
Supplement: Supplementary file 1 — Supplementary Material 1 [file 12879_2026_13369_MOESM1_ESM.docx]

**Supplementary Material**


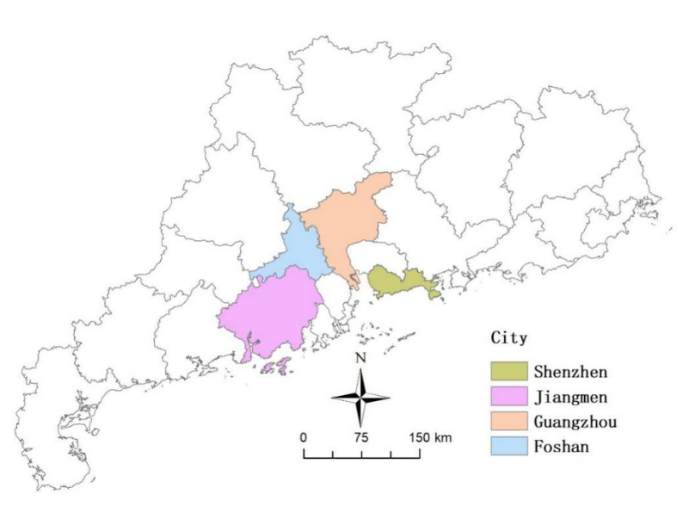


Figure S1. Geographical locations of the four cities of Guangzhou, Shenzhen, Foshan and Jiangmen


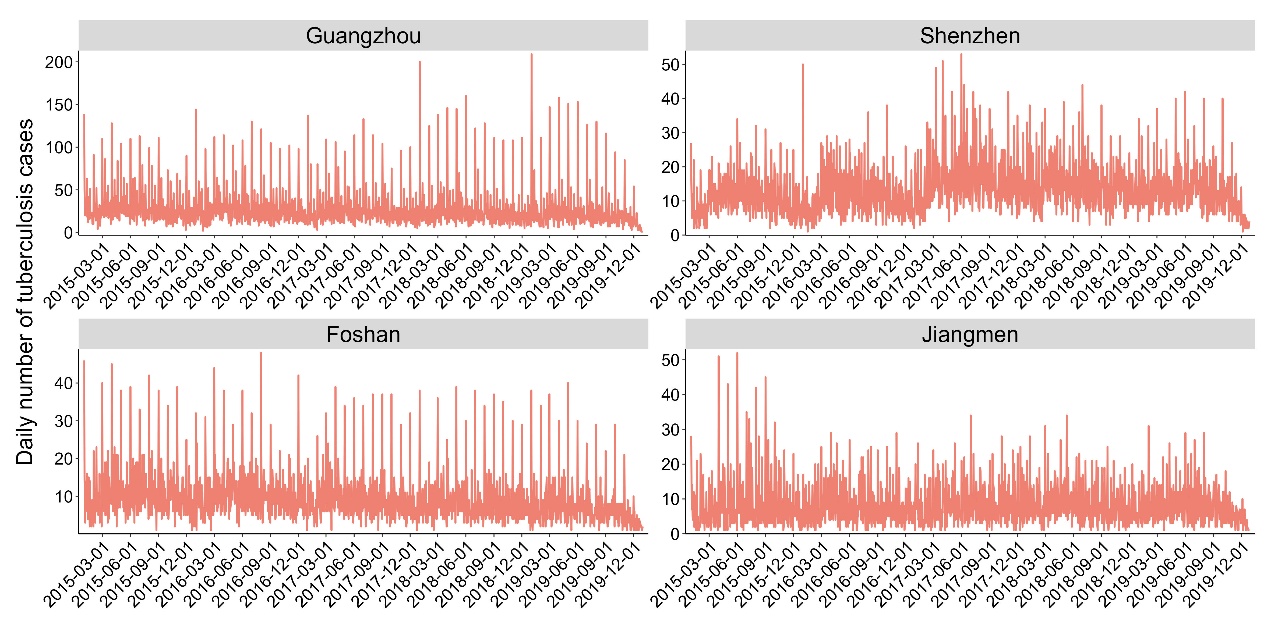


Figure S2. Daily number of tuberculosis cases in four cities from 2015 to 2019

Table S1. Assessment of multicollinearity among air pollutants using variance inflation factors (VIF)

| Particulate matter | SO_2_ | NO_2_ | O_3_ | CO |
| --- | --- | --- | --- | --- |
| PM_10_ | 1.99 | 3.69 | 1.65 | 2.08 |
| PM_2.5_ | 2.00 | 3.17 | 1.51 | 2.29 |

Table S2. *P*-values ​​for the non-linear test of the relationship between particulate matter and tuberculosis incidence risk in four cities

| City | PM_10_ | PM_2.5_ |
| --- | --- | --- |
| Guangzhou | <0.01 | <0.01 |
| Shenzhen | 0.18 | 0.09 |
| Foshan | 0.06 | 0.07 |
| Jiangmen | <0.01 | <0.01 |
